# Real-time monitoring of serotonin with highly selective aptamer-functionalized conducting polymer nanohybrids

**DOI:** 10.1186/s40580-022-00325-7

**Published:** 2022-07-12

**Authors:** Seong Gi Lim, Sung Eun Seo, Seon Joo Park, Jinyeong Kim, Yejin Kim, Kyung Ho Kim, Jai Eun An, Oh Seok Kwon

**Affiliations:** 1 Infectious Disease Research Center, Korea Research Institute of Bioscience and Biotechnology (KRIBB), Daejeon, 34141 Republic of Korea; 2 Department of Civil and Environmental Engineering, Yonsei University, Seoul, 03722 Republic of Korea; 3 Department of Biotechnology (Major), University of Science & Technology (UST), 125 Gwahak-ro, Yuseong-gu, Daejeon, 34141 Republic of Korea

**Keywords:** Serotonin, Aptasensor, Conducting polymer, Nanohybrids, FET

## Abstract

Adequate serotonin levels are pivotal to human well-being; thus, serotonin can be used as a biomarker because it regulates a wide range of physical and psychological functions. As an imbalance of serotonin is highly likely to initiate the pathogenesis of various disorders, monitoring serotonin levels in real time is in high demand for the early detection of disease. We fabricated a field-effect transistor (FET) biosensor based on aptamer-immobilized conducting polymer nanohybrids, which showed an instantaneous response toward serotonin in solution. The mechanism of serotonin detection was based on aptamer deformation after aptamer-ligand interaction and the consequential decrease in the charge carrier density of the FET template. Docking simulations with AutoDock/Vina and PyMOL were successfully used to investigate the binding site of serotonin in the loop structure of the aptamer. The fabricated FET template showed high sensitivity toward serotonin in the range of 10 fM to 100 nM, and the limit of detection (LOD) was exceptionally low at 10 fM. Moreover, the selectivity toward serotonin was confirmed by observing no signal after the injection of structural analogs, functional analogs and excess physiological biomolecules. The potential clinical application of this sensor was confirmed because it remained consistent when the buffer solution was exchanged for artificial serum or artificial cerebrospinal fluid (CSF). ^†^ S.G.L. and S.E.S. contributed equally to this work.

**Supplementary Information:**

The online version contains supplementary material available at 10.1186/s40580-022-00325-7.

## Introduction

Serotonin or 5-hydroxytryptamine (5-HT) is a monoamine neurotransmitter and is considered as one of the most important neurotransmitters due to its influence in every regulatory process. Serotonin modulates most brain functions, gastrointestinal endocrine system, cardiovascular and pulmonary physiology, and behavioural functions such as mood, perception, reward, aggression, memory, sexuality, attention, appetite, and sleep-wake [1–4]. The normal range of serotonin concentration in human blood and cerebrospinal fluid was investigated as 101–283 ng mL^−1^ and 0.8–3.7 nmol L^−1^ [5, 6]. Due to the regulative leverage of serotonin [4–12], an imbalance in serotonin levels induces both physical and psychological malfunctions in our body, and concurrent outbreaks prevail [13–16]. Lack of serotonin has the potential to cause major depression [17], a chronic psychiatric disorder with considerable prevalence triggered by abnormalities in the serotonin neurotransmission process. Excess serotonin possibly causes serotonin syndrome, an adverse drug reaction evoked by the simultaneous use of drugs in different classes [2, 17, 18].

High demands have been placed on the rapid detection of serotonin to pursue an accurate early diagnosis of several diseases [19, 20]. Currently, clinical diagnosis of major depression is dependent on symptoms that need to be reported by patients, which introduces a high possibility of misdiagnosis [17]. Although the most widely used treatments for the major depression are drugs regarding the serotonin system [17, 21–24], no studies have investigated the pathogenesis of depression in terms of serotonin levels and no laboratory criteria exist to confirm the case. Concerns are also increasing regarding serotonin syndrome due to its abundant presentation [18], as not only combinations of therapeutic drugs but also over-the-counter medicines or health supplements are possible suspects of pathogenesis [17, 25, 26]. The morbidity and mortality of serotonin syndrome are estimated to be high because ambiguity and inconsistency of symptoms make rapid recognition of the disease impossible [25–28]. However, technological advances [17] in laboratory tests have high potential to prevent the outbreaks of the diseases [28]. Currently, the level of serotonin is analysed using the classical method of liquid-chromatography/mass–spectrometry (LC/MS) after the pre-treatment of solid-phase extraction, which demands long analysis time, high expense, and professional skills for measurement.

Electrochemical sensors are in great interest as a promising technology to replace current methodologies of serotonin detection because they respond to serotonin simply and easily with high sensitivity and selectivity. Moreover, further applications in point-of-care industries are highly expected as they show instant responses toward serotonin [29–32]. A variety of technologies in electrical sensors have been developed for serotonin detection, such as voltammetry, chronoamperometry, and field-effect transistor [33–39]. For instance, the molecular imprinted sensor by Yola et al. [38] showed performance for serotonin with the limit detection of 200 fM in the presence of tryptophan, and the zinc oxide nanorod FET sensor produced by Sinha et al. [39] has wide linear range from 0.1 fM to 1 nM. However, methodologies based on redox reaction of the target molecule have limited physiological usability because serotonin shares the same electroactive moieties with the precursor molecules which exist in excess [40]; tryptophan, tryptamine, 5-hydroxytryptophan. Therefore, an ultraselective serotonin sensor platform with high sensitivity is demanded to distinguish serotonin from physiologically abundant biomolecules and to analyse serotonin deficiencies in medical samples after serial dilution.

Herein, we constructed a serotonin detection system to obtain a real-time response by combining a field-effect transistor (FET) with an aptamer that unravels when selectively interacting with serotonin [41]. For the substrate of FET system, nanohybrids coated with the carboxyl-group functionalized conductive polymer layer [42] were utilized to easily produce nanomaterials with desired properties in low price and to immobilize the aptamer molecules via formation of amide bonds. The serotonin detections of the fabricated FET system were monitored in real-time, and the high fidelity of the aptamer toward serotonin was discovered when compared with its precursors and other biomarkers [43]. The performance of the sensor was further demonstrated in physiological media to evaluate its efficacy, and its selective response toward serotonin was retained in artificial serum and artificial CSF [20, 44].

## Methods/experimental

### Materials and apparatus

3,4-Ethylenedioxythiophene (EDOT), polyacrylonitrile (PAN), dimethylformamide (DMF), ferric chloride, 4-(4,6-dimethoxy-1,3,5-triazin-2-yl)-4-methylmorpholinium chloride (DMTMM), hexamethyldisilazane (HMDS), 3,4-Dimethoxythiophene, *p*-toluenesulfonic acid monohydrate (pTsOH·H_2_O), butylated hydroxytoluene (BHT), tetrahydrofuran (THF), and Sylgard 184 polydimethylsiloxane (PDMS) were obtained from Sigma–Aldrich. Ethanol, toluene, methanol, hexane (Hex), hydrochloric acid (HCl), and ethyl acetate (EtOAc) were obtained from Samchun. Sodium hydroxide (NaOH) was purchased from Daejung. (S)-Methyl 2,3-dihydroxypropanoate (Methyl glycerate) was purchased from Combi-Block. AZ5214E was obtained from Clariant. A serotonin aptamer with the sequence 5′-CGACTGGTAGGCAGAT AGGGGAAGCTGATTCGAGCGTGGGTCG[C6 Amine]-3′ was synthesized by Bioneer. Phosphate buffered saline (PBS) pH 7.4 (1 ×) was purchased from Gibco™, and simulated blood serum and artificial CSF were purchased from Biochemazone. All reagents and solvents were used as received without further treatment.

### Synthesis of EDOT-Methyl ester

To a stirred solution of 3,4-Dimethoxythiophene (196 mg, 1.36 mmol) in toluene (15 mL) was added Methyl glycerate (816 mg, 6.80 mmol) under nitrogen. Then *p*-toluenesulfonic acid monohydrate (13 mg, 0.07 mmol) and Butylated hydroxytoluene (15 mg, 0.07 mmol) were added as catalysts. The reaction was stirred for 48 h under reflux at 100 ℃. The solvent was evaporated and the oily residue was purified by silica column chromatography using Hex:EtOAc 10:1 gradient mixtures to give product as a colorless oil (163 mg, 60%).

### Synthesis of EDOT-acid

To a solution of EDOT-Methyl ester (163 mg, 0.82 mmol) in THF (3 mL) and methanol (2 mL) was added 2 M NaOH (1 mL) at room temperature under nitrogen. After the reaction mixture was stirred 30 min the solvent was evaporated and diluted with EtOAc (30 mL) and, pH was adjusted 4 using 1 M HCl. Then washed with water (30 mL) twice. The separated organic layer was washed with the brine (15 mL), dried over Na_2_SO_4_, filtered and evaporated in vacuo to give product as a beige solid. (138 mg, 92%).

### Fabrication of the carboxylated PEDOT nanofiber

The solution for electrospinning was made by dissolving PAN in DMF for 12 h at 70 °C and was loaded in a 12 mL syringe with a 21-gauge needle. The solution was constantly added by a syringe pump at a steady rate of 1 mL h^-1^. Commercial aluminum foil, which was placed 15.5 cm apart, was used as the substrate, and a high voltage of 17 kV was applied by an electrospinning system (Nano NC). The prepared PAN nanofiber template was then dipped in 10 wt% ferric chloride in ethanol. After the removal of excess solution, the nanofiber template was transferred to a glass chamber sealed with a rubber septum. The chamber was kept under a constant pressure of 760 Torr and the monomer solution was injected at a constant ratio (EDOT: EDOT-acid = 71:1). For evaporating the liquid monomers, the chamber was heated to 60 °C, and the multidimensional conductive polymer was successfully fabricated with a thickness of 40 μm.

### Fabrication of the electrodes

A glass wafer was precleaned with HMDS to make the surface of the glass wafer hydrophobic for adsorbing the photoresist. The positive photoresist (AZ5214) was coated onto the substrate by a spin-coater with a thickness of 1.4 μm. An interdigitated microelectrode pattern was formed via ultraviolet (UV) exposure (light intensity = 23 mW) passing through a film mask using an aligner (MA-6 III, Karl-Suss). The exposed region was developed using AZ 300 MIF for 1 min with shaking. In the developed region, the source and drain electrodes (Cr/Au, 10 nm/160 nm thickness, respectively) were formed by an e-beam evaporator (ZZS550-2/D, Maestech). The wafer was immersed in acetone followed by sonication to remove the uncured photoresist. To connect the ends of interdigitated microelectrode, the fabricated PEDOT nanofiber template was applied as substrate. For coherence of the electrospun substrate, the nanofiber sheets were cut into 6 mm × 6 mm size and the resistence was measured by multimeter. Only those with resistence ca. 100 Ω were utilized, and a PDMS chamber with a 7 mm diameter was placed on the nanofiber sheet. Vacuum grease was used to physically anchor the substrate and chamber to the electrodes and to avoid leakage from the attached chamber. The chamber was filled with 40 µL of pH 7.4 PBS buffer or artificial physiological fluids as the electrolyte.

### Real-time sensing of serotonin utilizing the FET platform

The overall measurements were conducted using a Keithley 2612 A source meter that was connected to a probe station (MS-TECH, Model 4000 A). After setting the measurement range of the current change on the PEDOT nanofiber surface, the source-drain current of the sensor system was measured under a constant source-drain potential to the electrode (*V*_ds_). The system showed no signal when the electrolyte solution was applied to the chamber. Serotonin solution 1 mM dissolved in the PBS buffer was freshly made for every experimental trial as a stock solution and serially diluted with the electrolyte solution. 4 µL of diluted serotonin solutions were added into the chamber in sequence at constant time intervals. The response of each PEDOT-loaded electrode was collected, and following data were normalized according to the equation in Additional file 1: Fig. S6 to compare the current increase.

### Measurement of the target in the biofluids

Sensor performance for detecting serotonin and other interferents in body fluids (artificial serum and CSF) were measured using the same equipment as PBS diluted sample detection. The only difference was in the solutions for making the stock solution and the serially diluted solutions. Sensor responses were collected using body fluids instead of pH 7.4 PBS solution. Due to the excess of interfering biomolecules in the physiological environment, diluted standard solutions were also prepared in different concentrations, 100 fM serotonin and 1 mM other interfering substances.

### Instrumentation

Infrared (IR) spectra were obtained using a Fourier transform infrared (FT-IR) spectrometer (Nicolet iS50, Thermo Fisher Scientific Instrument). X-ray photoelectron spectroscopy (XPS) graphs were recorded utilizing a PHI 5000 VersaProbe instrument (Ulvac-PHI). Scanning electrode microscope (SEM) images were obtained using a S-4800 instrument (Hitachi). Fluorescence images were obtained using an EVOS M5000 instrument. The ^1^H NMR spectra was recorded on a Bruker AVANCE III HD (9.4 T) 400 spectrometer and High Resolution Mass Spectromter (HR-MS) was recorded on a Bruker Daltonik (micrOTOF-QII) at the Korea Advanced Institute of Science and Technology.

## Results and discussion

### Characterization of the FET substrate

Electrospun conductive polymer nanohybrids have been utilized as substrates for serotonin detection aptasensors. The overall fabrication process of the template is schematically illustrated in Fig. 2a. The facile fabrication of nanohybrids was initiated with electrospinning; the application of a high voltage to a viscous PAN solution induced the ejection of a nanofiber toward the collector. To achieve polymerization on the surface of electrospun nanofibers, they were immersed in a catalytic FeCl_3_ solution before coating. After removal of the initiator, in situ polymerization of EDOT-Acid/EDOT proceeded in a dry reactor with monomers in the vapor phase (Additional file 1: Fig. S1). The introduction of EDOT-Acid/EDOT onto the pure PAN nanofiber was crucial for two reasons: increased conductivity [45] and carboxyl group functionalization. Although pristine PAN nanofibers are nonconductive polymers, the addition of conductive substances easily provides the inert nanofibers with desirable electrochemical properties [45, 46]. PEDOT was utilized as an electrode coating due to its high conductivity, capacitance and thermodynamic stability [47, 48]. Moreover, a carboxyl group was introduced on the conductive polymer to form amide bonds with the biomolecule. The strategy to attain the required functional group was to intermingle the EDOT monomer solution with a trace amount of carboxyl-functionalized monomers (Additional file 1: Fig. S2, S3, S4) [49]. Consequently, vapor-phase polymerization was used to synthesize the p-type semiconductor nanohybrids, which were available for aptamer immobilization.

The comparison between the XPS spectra of the pristine PAN nanofibers and nanohybrids after vapor-phase polymerization showed the modified composition of the surface in detail. The C 1s XPS spectra are shown in Fig. 2b, and the large peak in common at 284.4 eV corresponded to *sp*^*3*^ C in the C–C or C–H bond [50]. A distinctive peak for pristine PAN nanofibers at 286.5 eV was assigned to *sp* C in nitrile bonds [50]. The spectra of the PAN/PEDOT nanohybrids had peaks at 286.5 and 288.8 eV, each of which was assignable to the ether groups in the EDOT molecules and carboxyl functional groups introduced on the surface, respectively [50]. The introduction of carboxyl groups on the substrate surface via polymerization was further confirmed with the O 1s XPS spectra, as shown in Fig. 2d. No peak was observed when the PAN nanofiber spectrum was analysed, while two distinctive peaks appeared at 531.59 eV and 533.08 eV in the PAN/PEDOT nanohybrids spectrum. The former peak with less binding energy was assigned to oxygen in the acyl group, C=O, and the other corresponded to oxygen forming a single bond with carbon, C–O [50]. Therefore, the production of carboxyl functional groups was concluded because of the congruent tendency from the C 1s and O 1s XPS spectra analysis. Figure 2c shows the N 1s spectrum of each templates. The distinctive peak of the nitrile bond at 398.5 eV was only observed in the PAN nanofiber spectrum [50]. The formation of the PEDOT layer was additionally confirmed because the S 2p spectrum of the PAN/PEDOT nanohybrids in Fig. 2e was consistent with thiophene 2p_3/2_ at 164.13 eV and 2p_1/2_ at 165.38 eV [51, 52].

Scanning electron microscopy (SEM) images were used to compare the morphological difference at the microscale after polymerization (Fig. 2f). From left to right, each corresponds to the pristine PAN nanofibers and carboxyl-EDOT/EDOT functionalized nanohybrids, respectively. Since the discrimination of the asperity between the pristine PAN film and PEDOT-coated PAN film was difficult [45], the protruding spike structures were induced from the introduction of carboxylic acid group. Properties that led to higher sensor performance, such as an enlarged surface area or increase in the number of active sites, were anticipated from the hierarchical structure on the surface of the modified nanofibers [53].

Fourier transform infrared (FT-IR) spectroscopy was utilized for the surface analysis of PEDOT-polymerized nanohybrids, and the corresponding data are shown in Fig. 2g. The pristine PAN nanofiber exhibited transmittance peaks of nitrile bonds at 2200 and 2855 cm^−1^, which represent the triple bonds between carbon and nitrogen and the stretching mode of N–H bonds, respectively [54, 55]. The existence of carboxyl groups was confirmed because the transmittance peaks at 1793 and 1751 cm^−1^ were attributed to the double bonds of carbonyl functional groups [56, 57]. The peak at 1522 cm^−1^ corresponded to the specific band stretching of the alkene group, which resulted from the double bonds in thiophene that was present in the monomer structure [56, 58].

### Characterization of the serotonin-binding aptamer immobilized on the FET substrate

An aptamer that binds to serotonin exclusively was anchored on the transistor substrate for selectively detecting the target biomolecule. Aptamer immobilization was established by a DMTMM coupling reaction, which consisted of amide bond formation between the carboxyl group of the nanohybrids and the primary amine of the aptamer and utilized DMTMM as the condensing agent [59, 60]. The scheme of the surface modification process is shown in Fig. 3a. The first step of the coupling reaction was the activation of the carboxyl groups on the surface via the introduction of aqueous DMTMM solution. After removal of the condensing agent residue, the bioprobe solution was treated. The lingering DMTMM fragments were substituted into the aptamer, and the amine group in the DNA sequence participated in amide bond formation; at this point, the coupling reaction was completed.

Optical and electrochemical techniques were used to confirm aptamer immobilization on the nanohybrids. Figure 3b shows the comparison of fluorescence between two substrates treated with aptamer solutions, one consisting of only nucleotides (left) and the other connected to the labelling agent (right). The latter was linked with 5′-fluorescein phosphoramidite (6-FAM), and the corresponding transistor template showed green fluorescence with light from 460 to 500 nm, while no emission was remarked from the counterpart. In Fig. 3c, the *I*-*V* curve has a linear current increase in the range from − 3 V to 3 V, which indicates fluent carrier mobility and the presence of ohmic contact of the fabricated electric system. The investigation of the *I*-*V* curve exhibited the electrical conductivity of the fabricated transistor platform and the absence or very low presence of a voltage drop across the interface was perceived between the metal electrode and nanofiber template. *I-V* curve was only presented with those samples coated with PEDOT which gave conductivity to the transistor platform. Despite no impediment in carrier movement was observed at the interface after aptamer immobilization, a decrease in conductivity was detected.

In Fig. 3d, the specific P 2p XPS spectra demonstrated the distinction between substrates, which occurred depending on the presence of the aptamer. While no peaks were observed for the nanohybrids without biomolecules, the surface-modified template showed peaks at 133.5 and 134.5 eV. These peaks were assigned to P 2p_1/2_ and 2p_3/2_, respectively, and represented the phosphate backbone structure of DNA [61–64]. The FT-IR spectra of the fabricated sensor platforms are shown in Fig. 3e. The vibrational band of the DNA-composing structure was detected at 1243.26 cm^−1^, which was assigned to be the asymmetric stretching of the phosphate group in the phosphodiester backbone of DNA [65, 66]. The additional peak observed at 3669 cm^−1^ was assigned to be an overtone of the carbonyl band with a bathochromic shift. Therefore, the modification of the transistor template was confirmed based on the two distinct spectrometry, which congruently indicated the phosphodiester bonds in the aptamer.

### Detection of serotonin with the FET-based sensor platform

**Fig. 1:**
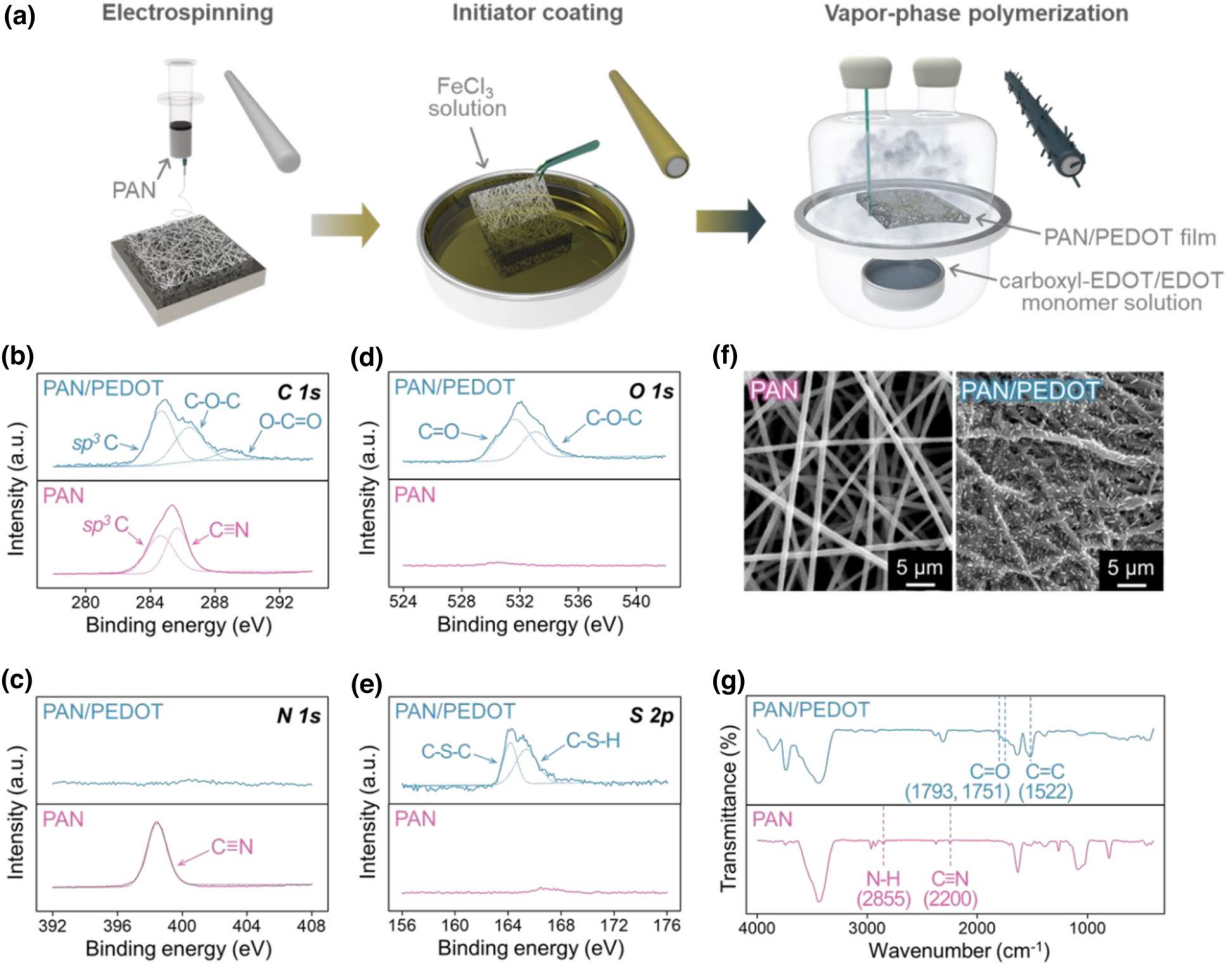
Fabrication of the carboxyl-functionalized PAN/PEDOT nanohybrids. **a** Schematic illustration of the nanofiber fabrication process. **b** C 1s, **c** N 1s, **d** O 1s and **e** S 2p spectra of the PAN nanofiber and PAN/PEDOT nanohybrids. **f** SEM image of the pristine PAN nanofiber and the carboxyl functionalized nanohybrids. **g** FT-IR data of the PEDOT nanohybrids containing carboxyl functional groups on their surface

The aptamer-immobilized PEDOT nanohybrids was loaded on an interdigitated microelectrode (IME) substrate as a transistor for the fabrication of an FET-based chemiresistive aptasensor platform (Fig. 1a). As shown in the schematic illustration, single-stranded DNA oligonucleotides formed functional three-dimensional structures that specifically bound to their target molecule with high affinity [41]. The interaction between the aptamer and ligand occurred after the addition of serotonin into the chamber filled with electrolyte. The target molecule occupied the inner ring structure of the aptamer [41, 67]. The formation of the aptamer-ligand complex induced a conformational change of the aptamer [41, 44, 68]. When the stem-loop part of the aptamer captured serotonin, the curved shape was stretched and reoriented [69]. To explore the formation mechanism of the aptamer-serotonin complex, ligand docking and binding site analysis were conducted with AutoDock/Vina and PyMOL (Fig. 1b) [70]. The aptamer formed a stable hydrogen bond with serotonin with a distance of 2.7 Å and a bond angle of 127°. Specifically, a strong secondary bond was constructed between the hydrogen in the secondary amine of the serotonin and the oxygen of the phosphate group, which was placed between the thymine and adenine of the loop structure [71]. In the absence of the ligand, there was no structural rearrangement of the aptamer.

The fabricated FET-based transducer was evaluated for its real-time response toward serotonin by measuring the drain-to-source current (*I*_ds_) after the serial injection of diluted serotonin. The performance of the sensor is shown in Fig. 1c. In previous research, the primary charge carrier of the carboxylated PEDOT nanohybrids was clearly defined as holes (h^+^) with p-type transistor characteristics. With the conformational change of the aptamer, the distance between the aptamer, which has a negative electric effect with the backbone, and the surface of the transistor increased. Consequently, holes accumulated on the surface of the PEDOT nanohybrids substrate [72, 73]. Higher amounts of serotonin result in a higher increase in the change in current. The real-time change in the source-drain current (Δ*I*/*I*_0_)_ds_ was compared at fixed values of applied voltage (a constant source-drain bias of *V*_ds_ = − 10 mV and a constant gate bias of *V*_g_ = 0.4 V) between the bare PEDOT nanohybrids as the corresponding control experiment and the aptamer-immobilized electrode. The performance of the sensor was presented as the normalized change in current (Δ*I*/*I*_0_)_ds_. There was no significant change in current from the bare IME when the serially diluted serotonin solution was added. As the target solution was added, the change in signal was observed within 0.6 s. The sensing capability of our novel detection platform showed remarkable performance with a detection limit of approximately 10 fM, which is 20 times higher than that of a previously developed sensor (Additional file 1: Fig. S6) [38]. Within the range of 10 fM to 10 nM, the measured *I*_ds_ of the platform diminished with increasing concentrations of serotonin. Based on the electrochemical response current versus serotonin concentration, a calibration curve was plotted, fitting with the Langmuir adsorption isotherm (Fig. 1d) [74]. The corresponding plot showed that the response was proportional to serotonin concentrations over a range of 10 fM to 10 nM. The PEDOT nanohybrids-based aptasensor showed saturation at serotonin concentrations above 100 nM. The slope of the plot increased until the surface was saturated with the target.

In addition, to investigate the performance of the fabricated electrode, the selectivity was examined, including various interferent species, such as epinephrine (EP), norepinephrine (NE), ascorbic acid (AA), tryptophan (Trp), tryptamine (Trm), and 5-hydroxytryptophan (5-HTP). These materials were selected based on two criteria: their potential for forming complexes with aptamers due to having similar conformational structures (Trp, Trm, 5-HTP) or function as neurotransmitter (NE and EP) and being a bodily fluid component (ascorbic acid). As shown in Fig. 1e, the proposed sensor platform presented a remarkable increase in current change on the aptamer-immobilized PEDOT nanohybrids, showing its highly selective sensing performance after the addition of 100 fM serotonin. In contrast, all interferents in excess (1 mM) did not produce a response on the surface of the platform when added sequentially. These results showed that the fabricated sensor platform demonstrated highly selective and sensitive sensing performance to the target molecule.

### Analysis of serotonin in model samples

Serotonin is measured in blood (serum), saliva, and brain fluid (CSF), showing equivalent serotonin levels based on being part of a peripheral system or the central nervous system (CNS) [75]. Each of the serotonin levels does not reflect all the serotonergic systems [76]. Furthermore, there is no correlation between platelet serotonin levels and CSF or between salivary serotonin levels and CSF [76]. For this reason, the detection of serotonin levels in each viscera is an independent parameter of the total amount of serotonin in the human body. In addition, each level of serotonin that is generated by other viscera possesses different roles [77, 78]. For example, serotonin in the CSF represents the concentration generated by neurotransmitters, and the serotonin level in blood represents the serotonin level generated by gut cells. Herein, for the further examination of the practicality of our sensor platform, the real-time measurement of serotonin included in the model sample (simulated bodily fluid: artificial serum and artificial CSF) was conducted with the same experimental conditions used with PBS buffer (Fig. 4a). The standard target molecule was diluted with the artificial bodily fluids. There was no change in current by the components of the artificial serum in the determination of serotonin with the developed PEDOT nanohybrids-based sensor. For monitoring other major amounts of serotonin in bodily fluid, serotonin was measured in CSF. The performance of the fabricated platform showed a uniform detection ability. The serotonin level included in those biofluids also showed the stable sensing performance of the sample compared to the sample diluted with PBS.

In addition, a selectivity test with simulated bodily fluid was also conducted to confirm the interfering effect of complex matrices (Fig. 4b). Each body fluids containing interfering species was injected individually to the platform and investigated the change of the current from the baseline current. The change in current was normalized based on the serotonin signal. The serotonin current was set as the standard value, and the other values were compared to the reference value. Among the interferent species, none of them showed noticeable signals on the sensor platform, showing normalized values of 0.012 (EP), 0.009 (NE), 0.02 (AA), 0.009 (Trp), 0.022 (Trm), and 0.029 (5-HTP) compared to the value of 1 (serotonin). The overall results are summarized with a bar graph for comparison. Therefore, serotonin was successfully detected in artificial serum and CSF compared to the interferents. In comparison with conventional diagnostic technologies of serotonin, the stable detection performance in the artificial body fluids and rapid derivation of results showed highly remarkable potential of the newly developed detection platform in the application of real-time monitoring of the change of serotonin level in human body (Additional file 1: Fig. S7).

Further investigation of the biosimulations confirmed that no structural changes occurred when the aptamer bound to the interfering species, and that the binding between the aptamer and serotonin was not interfered by other biomolecules. As shown in Fig. 4c, serotonin was the only molecule that bound to the aptamer within the loop structure. The precursor molecules of serotonin, Trp, 5-HTP, and Trm, were bound to the loop structure, but they must be bound to the inside of the loop structure in order to cause structural change. Other interfering species also bound to the aptamer, however, the binding sites were not associated with the loop structure. Therefore, the manufactured sensor system succeeded in selectively responding to serotonin even in the presence of other biomolecules.

## Conclusions

In summary, we developed a field-effect transistor-based biosensor to conduct the real-time monitoring of serotonin in body fluids. The selective target responsive aptamer was successfully immobilized on the surface of carboxyl-functionalized PAN/PEDOT nanohybrids and utilized as a channel layer of the transistor. To confirm the performance and characteristics of the developed sensor platform, various analytic methodologies were conducted. Compared to previous studies on serotonin sensors, our novel sensor platform was demonstrated to have the lowest detection limit of 10 fM and showed good selectivity for serotonin among various interferents. In addition, this study provided the specific binding site of target molecule to the aptamer utilizing biosimulation. Moreover, the detection of serotonin was conducted in several biological fluids, and the results indicated the potential of this sensor for application with real human samples. There were negligible effects on the sensing performance of serotonin from other biomaterials. The proposed sensor platform showed excellent performance under various conditions and demonstrated significant suitability for determining serotonin levels in an in vitro model that represented its potential clinical application.

.

**Fig. 2:**
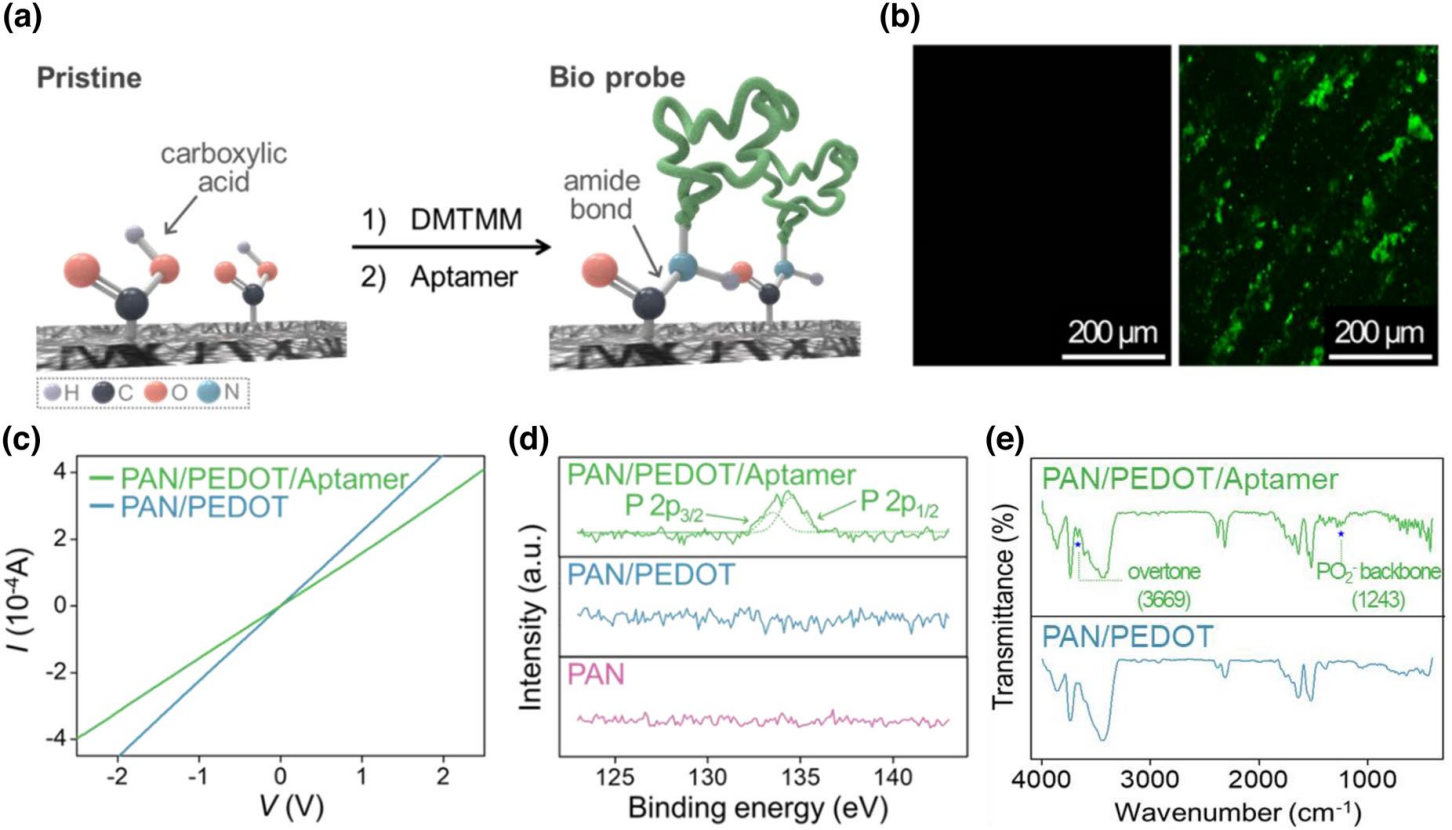
Characteristics of the aptamer-immobilized serotonin sensor. **a** Schematic illustration of the aptamer-immobilized electrode. **b** Fluorescence images of the surface-modified films with aptamers (left: aptamer alone; right: fluorescent probe-linked aptamer). **c** I-V curve of the surface-immobilized serotonin detection sensor platform. **d** XPS data with a P 2p peak, demonstrating aptamer attachment on the PEDOT film. **e** FT-IR data of the PEDOT nanohybrids after aptamer immobilization

**Fig. 3:**
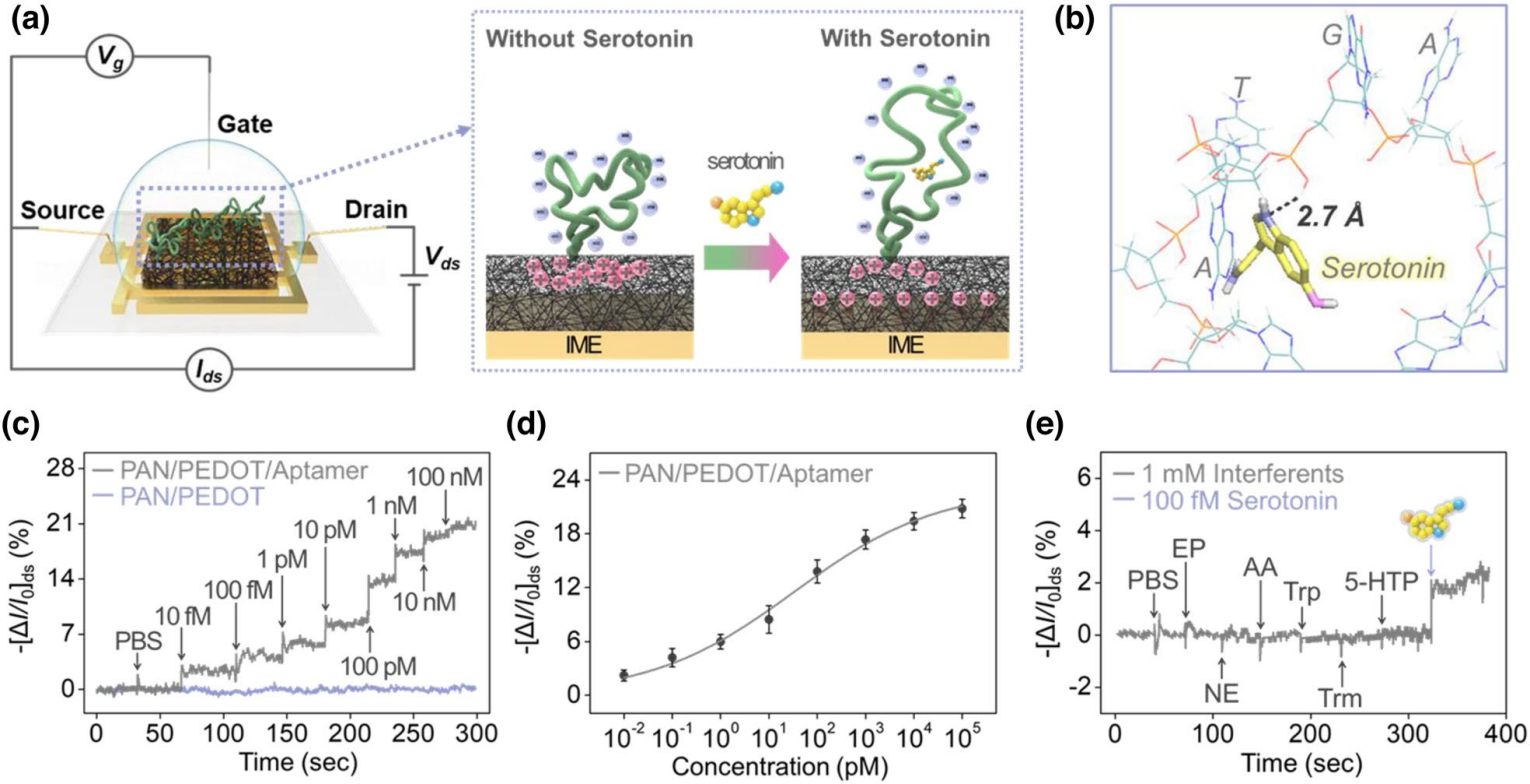
Electrical measurement of the PEDOT nanohybrids-loaded sensor platform. (a) Schematic illustration showing the device configuration of the PEDOT-based sensor platform (left) and the sensing mechanism between the aptamer and serotonin (right). (b) Biosimulation of the reaction between the serotonin molecule and aptamer. (c) Real-time measurement of serially diluted serotonin concentrations (range of 10 fM to 100 nM) with the bare electrode and aptamer-immobilized PEDOT nanohybrids-loaded electrode. (d) Calibrated response curve of the aptamer-immobilized transistor toward serotonin. (e) Selectivity results with 100 fM serotonin and 1 mM various interferents

**Fig. 4:**
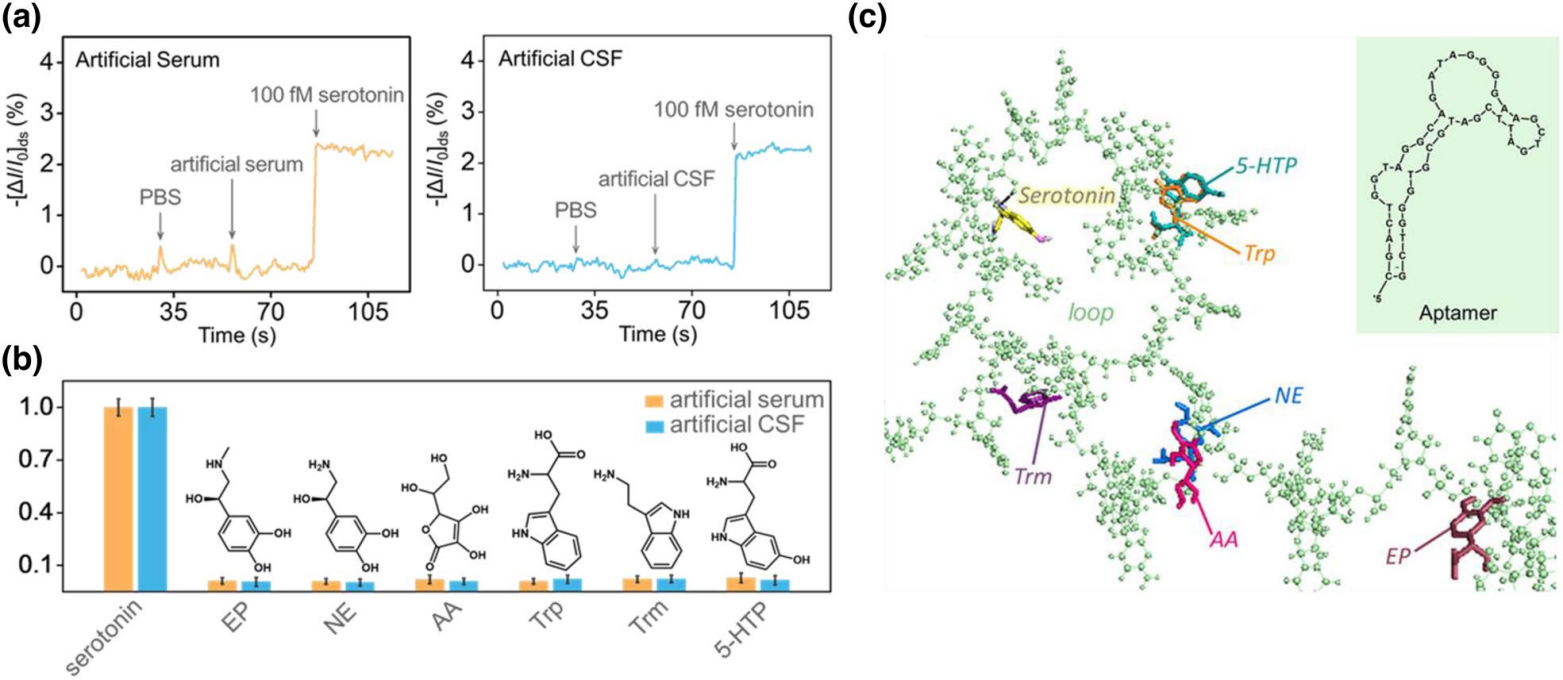
Sensing data of artificial biological fluids. Real-time sensing data of serotonin included in (**a**) artificial serum (left) and artificial CSF (right). **b** Bar graph of the selectivity test with artificial biofluids (artificial serum and CSF). **c** Molecular docking simulation of the aptamer and ligands, and the structure of the aptamer

## Supplementary information

**Additional file 1: Figure S1**. Optical images of (a) pristine PANnanofiber and (b) carboxyl-EDOT functionalized nanofiber. Figure S2. Synthetic scheme for EDOT-Acid. **Figure S3**. ^1^H-NMRof EDOT-Methyl ester in CDCl_3_. **Figure S4**. ^1^H-NMRof EDOT-Acid in DMSO. **Figure S5**. Equation for factor calculation. **Figure S6**. Table of comparing various sensor platforms forthe detection of serotonin. **FigureS7**. Real-time measurement of serotonin in the CSF solution with interferentmolecules.

## Data Availability

The datasets used and/or analysed during the current study are available from the corresponding author on reasonable request.
