# Supplementary material for: Real-time monitoring of serotonin with highly selective aptamer-functionalized conducting polymer nanohybrids
Source: Nano Converg. 2022 Jul 12;9:31. doi: 10.1186/s40580-022-00325-7 (PMC9279540; doi:10.1186/s40580-022-00325-7)
Supplement: Supplementary file 1 — Additional file 1: Figure S1. Optical images of (a) pristine PANnanofiber and (b) carboxyl-EDOT functionalized nanofiber. Figure S2. Synthetic scheme for EDOT-Acid. Figure S3. 1H-NMRof EDOT-Methyl ester in CDCl3. Figure S4. 1H-NMRof EDOT-Acid in DMSO. Figure S5. Equation for factor calculation. Figure S6. Table of comparing various sensor platforms forthe detection of serotonin. FigureS7. Real-time measurement of serotonin in the CSF solution with interferentmolecules. [file 40580_2022_325_MOESM1_ESM.doc]

Supporting Information

**Real-time monitoring of serotonin with highly selective aptamer-functionalized conducting polymer nanohybrids**

Seong Gi Lim^1, †^, Sung Eun Seo^1, 2, †^, Seon Joo Park^1^, Jinyeong Kim^1^, Yejin Kim^1^, Kyung Ho Kim^1^, Jai Eun An^1^, Oh Seok Kwon^1, 3, *^

^1^ Infectious Disease Research Center, Korea Research Institute of Bioscience and Biotechnology (KRIBB), Daejeon 34141, Republic of Korea.

^2^ Department of Civil and Environmental Engineering, Yonsei University, Seoul 03722, Republic of Korea.

^3^ Department of Biotechnology (Major), University of Science & Technology (UST), 125 Gwahak-ro, Yuseong-gu, Daejeon 34141, Republic of Korea.

^§^ S.G.L. and S.E.S. contributed equally to this work

*To whom correspondence should be addressed:

Prof. O.S. Kwon: Tel: +82-42-879-8284; Fax: +82-42-879-8594; E-mail: oskwon79@kribb.re.kr

**Optical images of pristine PAN nanofiber and carboxyl-EDOT functionalized nanofiber (Figure S1) .................................................................................................................................S3**

**Synthetic scheme for EDOT-Acid. (Figure S2) ........................................................................S4**

**^1^H-NMR of EDOT-Methyl ester in CDCl_3_ (Figure S3) ...........................................................S5**

**^1^H-NMR of EDOT-Acid in DMSO (Figure S4) .......................................................................S6**

**Equation for factor calculation (Figure S5) .............................................................................S7**

**Table of comparing various sensor platforms for the detection of serotonin (Figure S6) ...S8**

**Real-time measurement of serotonin in the CSF solution with interferent molecules (Figure S7) .................................................................................................................................................S9**

**
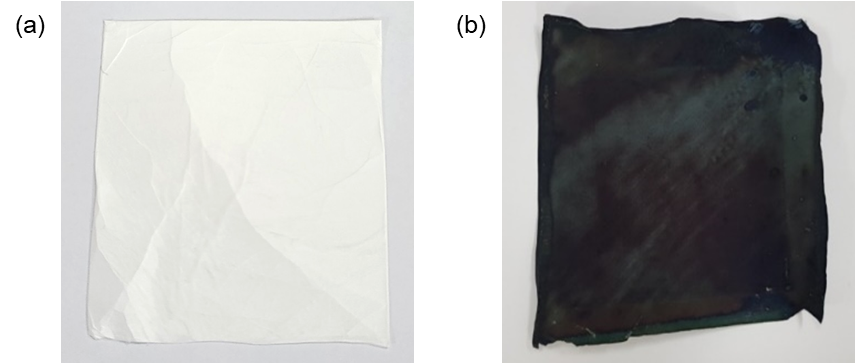
**

**Figure S1. Optical images of (a) pristine PAN nanofiber and (b) carboxyl-EDOT functionalized nanofiber.** The optical images in Fig. 1S demonstrated the color alteration from white of pristine PAN nanofiber to bluish-black of carboxylated-EDOT/EDOT polymerized PAN film.


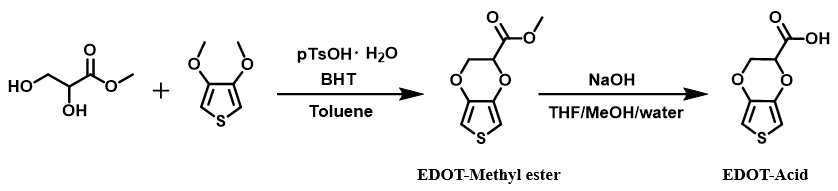


**Figure S2. Synthetic scheme for EDOT-Acid.**


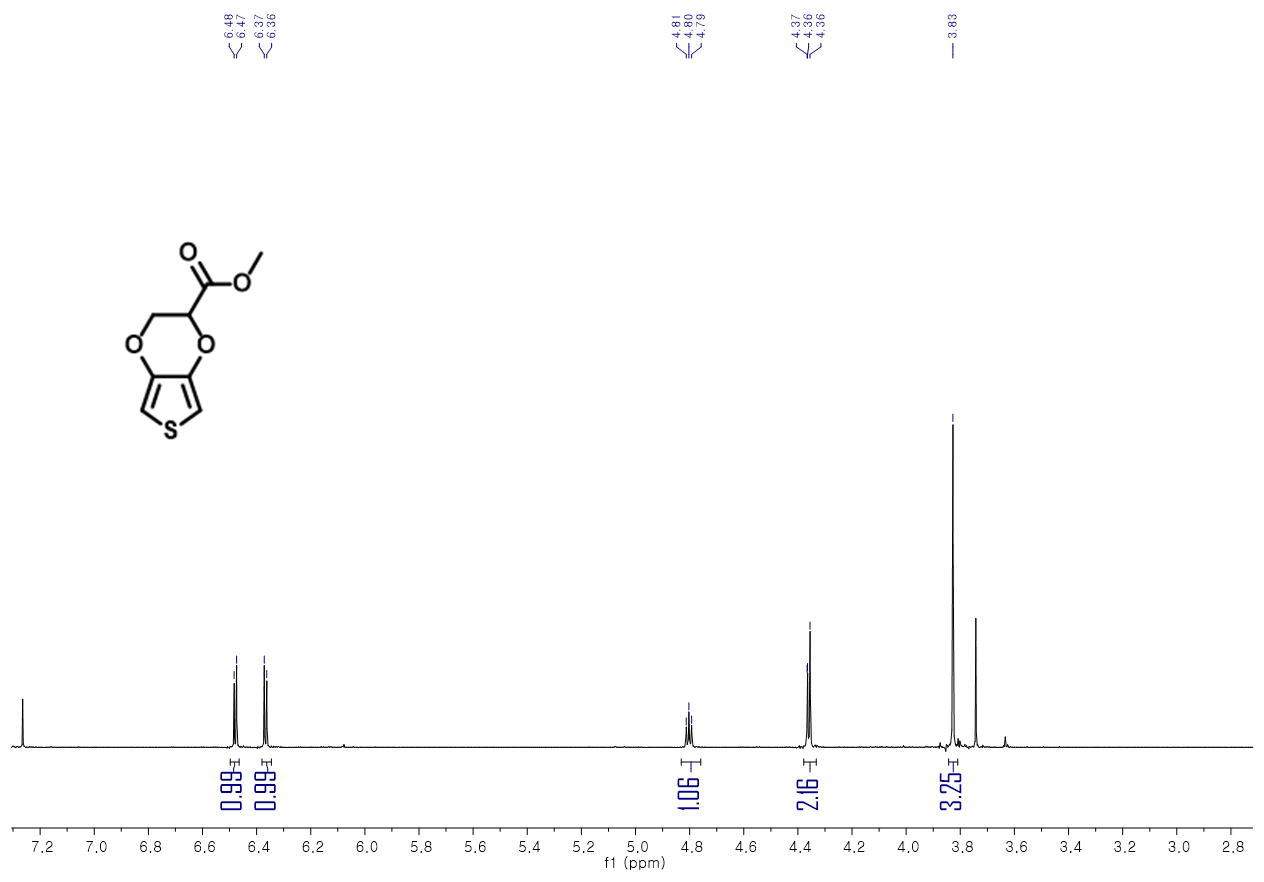


**Figure S3. ^1^H-NMR of EDOT-Methyl ester in CDCl_3_.** ^1^H NMR (400 MHz, CDCl3) δ 6.48 (d, J = 3.7 Hz, 1H), 6.37 (d, J = 3.7 Hz, 1H), 4.80 (t, J = 3.9 Hz, 1H), 4.38 – 4.33 (m, 2H), 3.83 (s, 3H)


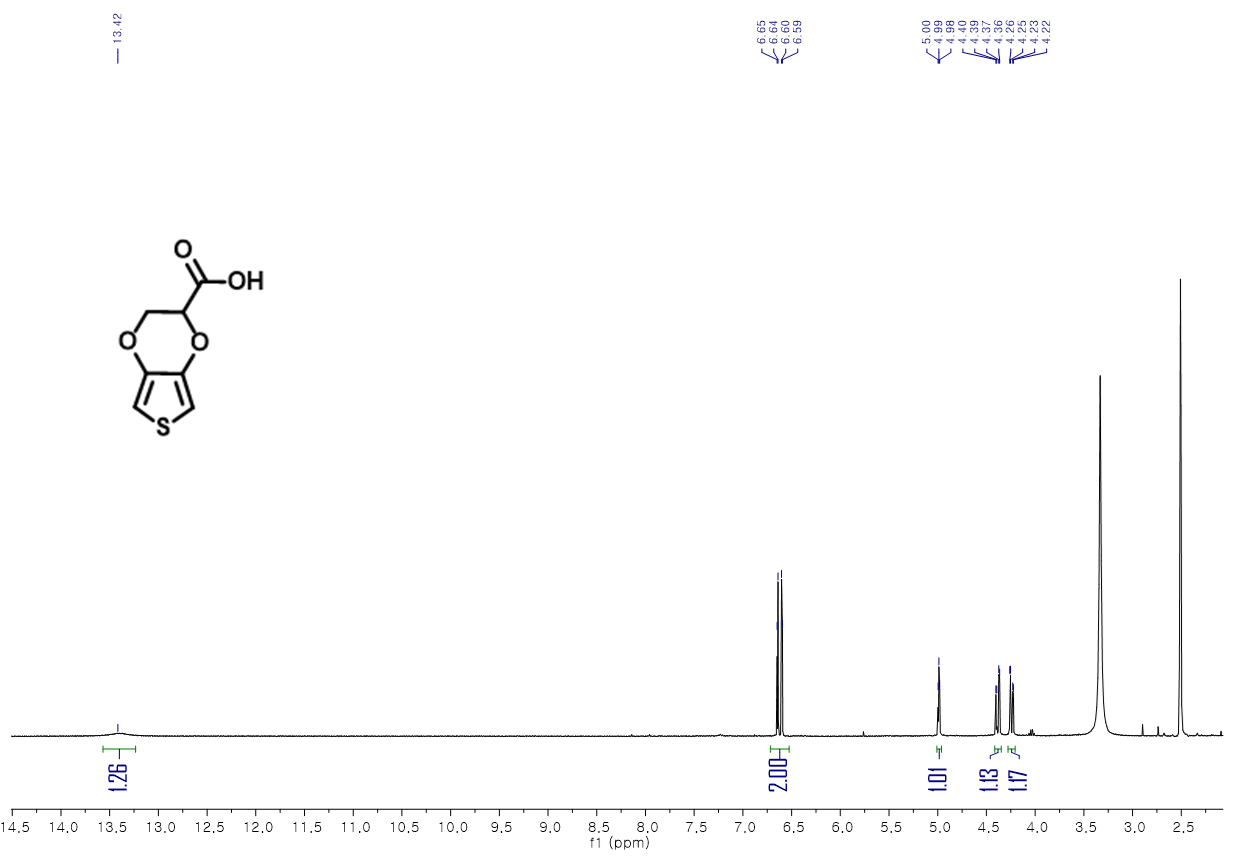


**Figure S4. ^1^H-NMR of EDOT-Acid in DMSO.** ^1^H NMR (400 MHz, DMSO-d6) δ 13.42 (s, 1H), 6.62 (dd, J = 18.2, 3.6 Hz, 2H), 4.99 (t, J = 3.2 Hz, 1H), 4.38 (dd, J = 11.9, 3.6 Hz, 1H), 4.24 (dd, J = 11.9, 2.8 Hz, 1H). ESI-MS (m/z) for C7H6O4S ^+^:00000, Calc.: 185.9987


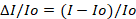
 *...............................................................* (a)


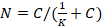
 ............................................................... (b)

**Figure S5. Equation for factor calculation.** (a) The normalization of the current intensity, where *I_o_* is the initial current and *I* is measured current. (b) Langmuir’s adsorption isotherm equation, where *C* is the concentration of ligand and *N* is the normalized sensitivity.

**
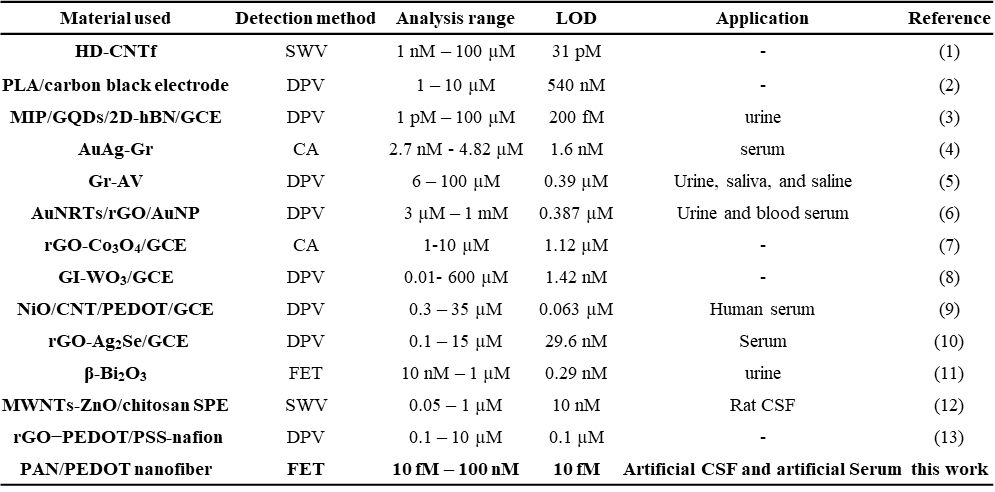
**

**Figure S6. Table of comparing various sensor platforms for the detection of serotonin.**


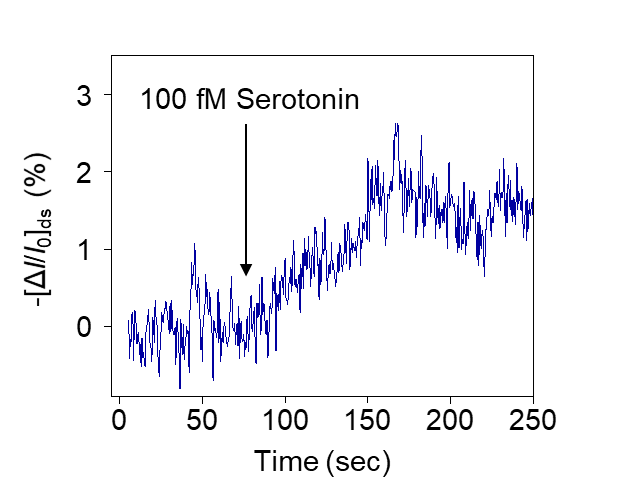


**Figure S7. Real-time measurement of serotonin in the CSF solution with interferent molecules.**

# References

1. P. Gupta, K. Tsai, C. K. Ruhunage, V. K. Gupta, C. E. Rahm, D. Jiang, N. T. Alvarez, Anal. Chem. **92**, 8536 (2020).
2. H. H. Hamzah, O. Keattch, M. S. Yeoman, D. Covill, B. A. Patel, Anal. Chem. **91**, 12014 (2019).
3. M. L. Yola, N. Atar, Appl. Surf. Sci. **458**, 648 (2018).
4. T. D. Thanh, J. Balamurugan, H. V. Hien, N. H. Kim, J. H. Lee, Biosens. Bioelectron. **96**, 186 (2017).
5. L. O. Orzari, R. Cristina de Freitas, I. Aparecida de Araujo Andreotti, A. Gatti, B. C. Janegitz, Biosens. Bioelectron. **138**, 111310 (2019).
6. K. Mahato, B. Purohit, K. Bhardwaj, A. Jaiswal, P. Chandra, Biosens. Bioelectron. **142**, 111502 (2019).
7. M. M. Shahid, P. Rameshkumar, A. Numan, S. Shahabuddin, M. Alizadeh, P. S. Khiew, W. S. Chiu, Mater. Sci. Eng. C*.* **100**, 388 (2019).
8. A. C. Anithaa, K. Asokan, C. Sekar, Sensors Actuators, B Chem. **238**, 667 (2017).
9. D. Sun, H. Li, M. Li, C. Li, H. Dai, D. Sun, B. Yang, Sens. Actuators B Chem. **259**, 433 (2018).
10. S. Panneer Selvam, K. Yun, Sens. Actuators B Chem. **302**, 127161 (2020).
11. S. Veeralingam, S. Badhulika, Sens. Actuators B Chem. **321**, 128540 (2020).
12. Y. Wang, S. Wang, L. Tao, Q. Min, J. Xiang, Q. Wang, J. Xie, Y. Yue, S. Wu, X. Li, H. Ding, Biosens. Bioelectron. **65**, 31 (2015).
13. W. Al-Graiti, J. Foroughi, Y. Liu, J. Chen, ACS Omega **4**, 22169 (2019).
